# Associations Between the Modified Cardiometabolic Index and Stroke in Patients With Different Glucose Metabolism Statuses: Evidence From a Nationally Representative Survey

**DOI:** 10.31083/RCM45989

**Published:** 2026-03-19

**Authors:** Tingting Deng, Guiling Wu, Xinghuan Liang, Yajuan Peng, Zhiyuan Dong, Yu Shen, Yingfen Qin

**Affiliations:** ^1^Department of Endocrinology, The First Affiliated Hospital of Guangxi Medical University, 530021 Nanning, Guangxi, China; ^2^Department of Endocrinology, The Fourth Affiliated Hospital of Guangxi Medical University, 545000 Liuzhou, Guangxi, China; ^3^Department of Neurology, The First Affiliated Hospital of Nanchang University, 330006 Nanchang, Jiangxi, China

**Keywords:** cardiometabolic risk factors, stroke, glucose metabolism, longitudinal studies, China

## Abstract

**Background:**

The association between the modified cardiometabolic index (MCMI) and the risk of incident stroke across patients with different glycemic statuses remains unclear. This study aimed to investigate the relationship between baseline MCMI levels and incident stroke in Chinese middle-aged and older adults with varying glucose metabolism states.

**Methods:**

Data were obtained from the China Health and Retirement Longitudinal Study (CHARLS) conducted in 2011, 2013, 2015, and 2018. Kaplan–Meier curves, multivariable Cox proportional hazards models, and restricted cubic spline analyses were employed to assess the relationship between the MCMI and stroke risk stratified by glycemic status. Subgroup and sensitivity analyses were performed to confirm the robustness of the findings.

**Results:**

A total of 7455 participants were included. A total of 457 individuals (6.13%) experienced stroke events during a median follow-up of 7 years. A significant linear association was observed between a higher MCMI and increased stroke risk. A nonlinear relationship was detected among participants with normal glucose regulation (NGR), with a sharp increase in risk beyond an MCMI threshold of 1.904 (hazard ratio (HR) = 1.85; 95% confidence interval (CI): 1.24–2.76; *p* = 0.003). An increased MCMI was also associated with increased stroke risk in individuals with prediabetes (HR = 1.34, 95% CI: 1.03–1.75) but not in individuals with diabetes. The associations varied across subgroups according to gender, residence, body mass index, and use of cardiovascular medications. Sensitivity analyses supported the stability of the results.

**Conclusion:**

An elevated MCMI is positively associated with incident stroke, particularly in individuals with NGR or prediabetes. Early identification of a high MCMI may be valuable for stroke prevention, risk stratification, and timely intervention in community populations.

## 1. Introduction

Stroke is an acute focal injury of the central nervous system caused by a vascular event, including ischemic stroke, intracerebral hemorrhage, and subarachnoid hemorrhage, and it remains one of the leading causes of disability and death worldwide [1, 2]. With the progressive aging of the population, China’s stroke burden has become the highest worldwide, and stroke is now the leading cause of death among the Chinese population [3, 4]. These trends highlight an urgent need for refined risk stratification tools and targeted prevention strategies to mitigate the growing public health burden.

The cardiometabolic index (CMI), which is calculated as the product of the waist-to-height ratio and the triglyceride-to-high-density lipoprotein cholesterol (TG/HDL-C) ratio, integrates markers of abdominal obesity and dyslipidemia and was initially used to identify patients with diabetes [5]. Owing to its accessibility and cost-effectiveness, the CMI has also been shown to be predictive of conditions such as hyperuricemia, metabolic-associated fatty liver disease, and hypertensive metabolic syndrome [6, 7, 8]. Notably, previous studies have demonstrated associations between CMI and stroke. Multiple studies from China have identified CMI as an effective predictor of stroke in middle-aged and older adults, and similar findings have been reported in Western populations, supporting its utility in predicting cardiovascular events in aging cohorts [9, 10, 11, 12, 13]. Diabetes is a well-established independent risk factor for stroke, with diabetic individuals facing a 2–4-fold higher risk of ischemic stroke than the general population [14]. Insulin resistance (IR), the core pathological feature of type 2 diabetes, has also emerged as an independent risk factor for stroke, beyond the effects of hyperglycemia [15, 16]. The triglyceride-glucose (TyG) index, a reliable biomarker of IR, has been validated in numerous studies for its association with stroke risk [17, 18, 19]. Therefore, developing a composite index that incorporates IR, abdominal obesity, and dyslipidemia could be crucial for improving stroke risk prediction.

The modified cardiometabolic index (MCMI), derived from both CMI and TyG, combines parameters of insulin resistance, abdominal obesity, and lipid abnormalities and has recently been proposed to be a predictor of hepatic steatosis and fibrosis [20]. However, despite growing interest, no study has explored the association between the MCMI and incident stroke in individuals with different glycemic statuses. To address this gap, we conducted a longitudinal analysis using data from the China Health and Retirement Longitudinal Study (CHARLS) to investigate the relationship between MCMI and stroke risk among Chinese middle-aged and older adults stratified by glycemic status, aiming to provide new insights for stroke prevention, early diagnosis, and control.

## 2. Methods

### 2.1 Study Design and Population

**Fig. 1.**
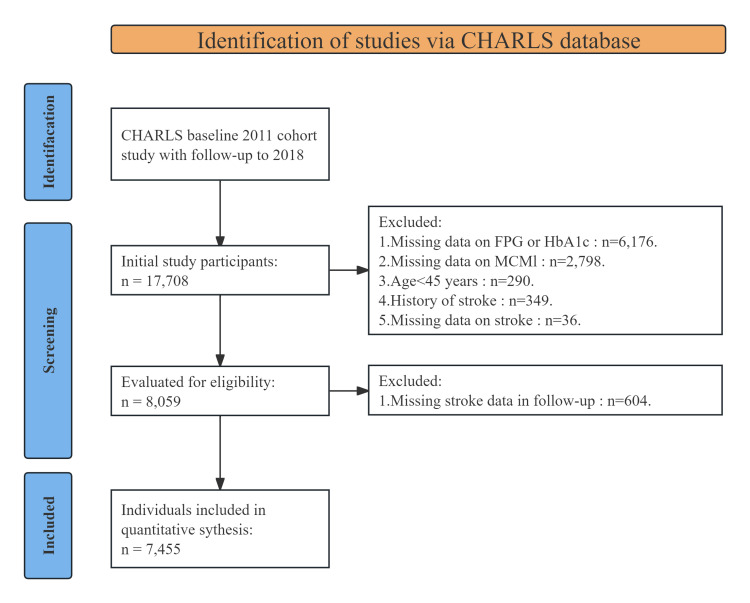
**Flowchart of patients included in the study**. CHARLS, China Health and Retirement Longitudinal Study; FPG, fasting plasma glucose; HbA1c, glycated hemoglobin; MCMI, modified cardiometabolic index.

The data were obtained from the China Health and Retirement Longitudinal Study (CHARLS) database, a nationally representative longitudinal survey that has collected health-related data on individuals aged 45 years and older in China since 2011. The specific waves used in this study and their corresponding links are as follows: (1) 2011 National Baseline Survey (https://charls.charlsdata.com/pages/Data/2011-charls-wave1/zh-cn.html), (2) 2013 National Follow-up Survey (https://charls.charlsdata.com/pages/Data/2013-charls-wave2/zh-cn.html), (3) 2015 National Follow-up Survey (https://charls.charlsdata.com/pages/Data/2015-charls-wave4/zh-cn.html). Participants are followed up with every two years through face-to-face computer-assisted personal interviews [21]. In this prospective cohort study, we analyzed data from the CHARLS baseline wave in 2011 through the follow-up in 2018. The initial cohort included 17,708 participants. Participants were excluded based on the following criteria: (1) implausible anthropometric measurements at baseline, including body mass index (BMI) <10 or >100 kg/m^2^ waist circumference (WC) <40 cm or >200 cm; missing essential demographic or biochemical variables (e.g., fasting glucose, triglyceride, HDL cholesterol levels) required for calculating the metabolic index CMI; (2) age <45 years at baseline or a self-reported history of stroke or unclear stroke history; or (3) missing stroke outcome data. The detailed inclusion and exclusion process is illustrated in Fig. 1.

### 2.2 Data Collection

During the baseline survey in 2011, trained personnel collected and recorded demographic information and health-related behaviors using standardized questionnaires. The collected data included gender, age, education level, marital status, smoking and alcohol consumption status, and medical history, including cardiovascular disease, stroke, kidney disease, dyslipidemia, hypertension, and the use of antihypertensive or lipid-lowering medications. Anthropometric measurements were also obtained, including height, weight, WC, and blood pressure, were also obtained. In addition, fasting venous blood samples were collected and processed for laboratory testing.

### 2.3 Indicator Calculation Formula

The MCMI was calculated using the following formula: MCMI = ln [TG (mg/dL) × fasting blood glucose (mg/dL) / HDL-C (mg/dL)] × WC (cm) / height (cm). In addition, the CMI was calculated as follows: CMI = TG (mg/dL) / HDL-C (mg/dL) × WC (cm) / height (cm) [21]. The TyG index was calculated using the formula: TyG = ln [TG (mg/dL) × fasting blood glucose (mg/dL) / 2]. BMI was calculated as body weight (kg) divided by height squared (m^2^). The waist-to-height ratio (WHtR) was defined as WC (cm) divided by height (cm).

### 2.4 Outcome Assessment and Follow-Up

The incidence of stroke during the follow-up period was determined based on self-reported data. Trained interviewers asked participants the following questions: (1) “Have you ever been diagnosed with a stroke by a doctor?” (2) “Since the last interview, have you been diagnosed with a stroke by a doctor?” and (3) “When were you first diagnosed with this condition, or when did you first become aware of it?” The time of stroke onset was defined by the participant’s response to question (3).

### 2.5 Definitions of Variables

Participants were categorized as underweight (BMI <18.5 kg/m^2^), normal weight (18.5–23.9 kg/m^2^), overweight (24.0–27.9 kg/m^2^), or obese (≥28.0 kg/m^2^) [22]. Hypertension and diabetes were defined by self-reported diagnosis, use of antihypertensive or antidiabetic medications, or clinical measurements. Diabetes was defined as fasting plasma glucose (FPG) concentration ≥126 mg/dL or a glycated hemoglobin (HbA1c) concentration ≥6.5%. Prediabetes was defined as an FPG between 100 and 125 mg/dL or HbA1c between 5.7% and 6.4%, while individuals not meeting either criterion were classified as having normal glucose regulation (NGR) [23, 24]. Dyslipidemia was defined as a self-reported diagnosis of abnormal lipid metabolism, current use of lipid-lowering medications, or any of the following laboratory criteria: total cholesterol (TC) ≥240 mg/dL, TG ≥150 mg/dL, HDL-C <40 mg/dL, or low-density lipoprotein cholesterol (LDL-C) ≥160 mg/dL [24, 25].

### 2.6 Statistical Analyses

Details of the missing data are provided in **Supplementary Table 1**. To enhance the robustness of the findings, MCMI was analyzed as a continuous variable and by quartiles: Q1 (MCMI ≤2.442), Q2 (2.442 < MCMI ≤ 2.867), Q3 (2.867 < MCMI ≤ 3.373), and Q4 (MCMI >3.373). Kaplan–Meier survival analysis was used to estimate the cumulative incidence of stroke across individuals in different MCMI quartiles, and differences were assessed using the log-rank test. Before multivariable modeling, we used the generalized variance inflation factor (GVIF) to assess potential multicollinearity among selected covariates [26]. Variables with a VIF >5 (TC and LDL-C) were excluded from the final Cox proportional hazards regression model (**Supplementary Table 2**). Multivariate Cox models were then applied to estimate hazard ratios (HRs) and 95% confidence intervals (CIs) for the association between MCMI and incident stroke in the overall cohort and across glycemic status subgroups. Receiver operating characteristic (ROC) curves were also constructed to evaluate the predictive value of the MCMI, CMI, WHtR, and TyG index for the incidence of stroke. The area under the curve (AUC) was used to compare the diagnostic efficacy of the MCMI with that of the CMI, WHtR, and TyG. To evaluate potential nonlinear associations, we used restricted cubic spline (RCS) regression models to assess the relationship between MCMI and stroke risk, overall and stratified by glycemic status. When significant nonlinearity was detected, a piecewise regression model was applied to identify inflection points using a recursive algorithm [27]. Subgroup analyses and interaction terms were incorporated to explore potential effect modification and heterogeneity. Additional sensitivity analyses were performed to ensure robustness: (1) reanalysis after complete case exclusion; (2) reanalysis after excluding deceased participants; (3) logistic regression analysis of MCMI and stroke incidence; and (4) calculation of the E value to estimate the potential influence of unmeasured confounders on the observed associations [28]. All the statistical analyses were performed using R software (version 4.2.2, Vienna, Austria).

## 3. Results

### 3.1 Participants

During the 7-year follow-up period, a total of 7455 participants were included, with a mean age of 58.80 years, 3419 of whom (45.86%) were male. The anthropometric and biochemical characteristics of participants stratified by MCMI quartiles are presented in Table 1. The results indicated that parameters such as BMI, WC, systolic blood pressure (SBP), diastolic blood pressure (DBP), FPG levels, HbA1c levels, TC levels, TG levels, C-reactive protein (CRP) levels, and BMI showed a significant upward trend with increasing MCMI levels. In contrast, the HDL-C level and blood urea nitrogen (BUN) level tended to decrease.

**Table 1.** **Demographic and baseline characteristics of the population**.

Characteristic		MCMI quartiles					*p*
		Overall	Q1	Q2	Q3	Q4	
No. of subjects		7455	1864	1864	1863	1864	
Age (year)		58.80 ± 9.08	58.65 ± 9.20	58.68 ± 9.12	58.65 ± 8.98	59.23 ± 9.01	0.142
Age							0.252
	45–59	4257 (57.10%)	1082 (58.05%)	1079 (57.89%)	1068 (57.33%)	1028 (55.15%)	
	60 and over	3198 (42.90%)	782 (41.95%)	785 (42.11%)	795 (42.67%)	836 (44.85%)	
Gender							<0.001
	Female	4036 (54.14%)	741 (39.75%)	969 (51.98%)	1073 (57.60%)	1253 (67.22%)	
	Male	3419 (45.86%)	1123 (60.25%)	895 (48.02%)	790 (42.40%)	611 (32.78%)	
Education							0.004
	Second school and over	2201 (29.52%)	558 (29.94%)	528 (28.33%)	591 (31.72%)	524 (28.11%)	
	Primary	3047 (40.87%)	793 (42.54%)	798 (42.81%)	715 (38.38%)	741 (39.75%)	
	Illiterate	2207 (29.60%)	513 (27.52%)	538 (28.86%)	557 (29.90%)	599 (32.14%)	
Marital status							0.627
	Married	6570 (88.13%)	1649 (88.47%)	1642 (88.09%)	1651 (88.62%)	1628 (87.34%)	
	Other	885 (11.87%)	215 (11.53%)	222 (11.91%)	212 (11.38%)	236 (12.66%)	
Location							<0.001
	Village	4876 (65.41%)	1356 (72.75%)	1309 (70.23%)	1157 (62.10%)	1054 (56.55%)	
	Community	2579 (34.59%)	508 (27.25%)	555 (29.77%)	706 (37.90%)	810 (43.45%)	
Drinking status							<0.001
	Never	5233 (70.19%)	1135 (60.89%)	1298 (69.64%)	1375 (73.81%)	1425 (76.45%)	
	Seldom	1046 (14.03%)	300 (16.09%)	268 (14.38%)	237 (12.72%)	241 (12.93%)	
	Often	1176 (15.77%)	429 (23.02%)	298 (15.99%)	251 (13.47%)	198 (10.62%)	
Smoking status							<0.001
	Never	4600 (61.70%)	927 (49.73%)	1131 (60.68%)	1218 (65.38%)	1324 (71.03%)	
	Former	607 (8.14%)	142 (7.62%)	142 (7.62%)	150 (8.05%)	173 (9.28%)	
	Current	2248 (30.15%)	795 (42.65%)	591 (31.71%)	495 (26.57%)	367 (19.69%)	
Stroke		457 (6.13%)	66 (3.54%)	106 (5.69%)	116 (6.23%)	169 (9.07%)	<0.001
Hypertension		3025 (40.58%)	493 (26.45%)	622 (33.37%)	822 (44.12%)	1088 (58.37%)	<0.001
Dyslipidemia		3537 (47.44%)	319 (17.11%)	585 (31.38%)	1028 (55.18%)	1605 (86.11%)	<0.001
Heart problem		835 (11.20%)	151 (8.10%)	165 (8.85%)	223 (11.97%)	296 (15.88%)	<0.001
Kidney disease		422 (5.66%)	125 (6.71%)	97 (5.20%)	103 (5.53%)	97 (5.20%)	0.149
GMS							<0.001
	NGR	3005 (40.31%)	1068 (57.30%)	890 (47.75%)	684 (36.71%)	363 (19.47%)	
	Prediabetes	3292 (44.16%)	695 (37.29%)	805 (43.19%)	907 (48.68%)	885 (47.48%)	
	Diabetes	1158 (15.53%)	101 (5.42%)	169 (9.07%)	272 (14.60%)	616 (33.05%)	
BMI status							<0.001
	Underweight	484 (6.49%)	352 (18.88%)	92 (4.94%)	29 (1.56%)	11 (0.59%)	
	Normal	3917 (52.54%)	1338 (71.78%)	1338 (71.78%)	856 (45.95%)	385 (20.65%)	
	Overweight	2192 (29.40%)	144 (7.73%)	405 (21.73%)	805 (43.21%)	838 (44.96%)	
	Obesity	862 (11.56%)	30 (1.61%)	29 (1.56%)	173 (9.29%)	630 (33.80%)	
Lipid-lowering drugs		359 (4.82%)	31 (1.66%)	47 (2.52%)	79 (4.24%)	202 (10.84%)	<0.001
Antihypertensive drugs		1382 (18.54%)	165 (8.85%)	231 (12.39%)	396 (21.26%)	590 (31.65%)	<0.001
Heart problem medications		550 (7.38%)	109 (5.85%)	111 (5.95%)	129 (6.92%)	201 (10.78%)	<0.001
BMI, kg/m^2^		23.55 ± 3.95	20.76 ± 3.03	22.31 ± 2.61	24.29 ± 3.19	26.83 ± 3.95	<0.001
WC, cm		84.30 ± 12.33	72.76 ± 13.48	81.67 ± 6.41	87.81 ± 7.25	94.99 ± 8.12	<0.001
SBP, mmHg		130.07 ± 21.23	124.66 ± 20.07	127.67 ± 20.72	131.17 ± 20.84	136.80 ± 21.36	<0.001
DBP, mmHg		75.59 ± 12.00	72.38 ± 11.50	74.54 ± 11.90	76.28 ± 11.69	79.17 ± 11.88	<0.001
FPG, mg/dL		109.49 ± 33.99	99.21 ± 17.12	102.41 ± 19.25	107.97 ± 26.65	128.39 ± 52.29	<0.001
HbA1c, %		5.28 ± 0.80	5.10 ± 0.46	5.14 ± 0.58	5.25 ± 0.65	5.63 ± 1.18	<0.001
TC, mg/dL		194.78 ± 38.77	187.05 ± 34.96	190.67 ± 36.08	194.90 ± 37.28	206.49 ± 43.46	<0.001
TG, mg/dL		131.17 ± 110.11	72.57 ± 33.17	96.73 ± 33.71	127.05 ± 50.27	228.35 ± 172.22	<0.001
LDL, mg/dL		117.59 ± 35.00	110.89 ± 30.40	118.37 ± 31.80	122.55 ± 33.38	118.56 ± 42.19	<0.001
BUN, mg/dL		15.71 ± 4.42	16.35 ± 4.65	15.68 ± 4.39	15.61 ± 4.42	15.18 ± 4.13	<0.001
Scr, mg/dL		0.78 ± 0.19	0.78 ± 0.17	0.77 ± 0.19	0.78 ± 0.19	0.77 ± 0.19	0.480
CRP, mg/dL		2.54 ± 6.92	2.29 ± 7.15	2.40 ± 7.18	2.60 ± 7.98	2.88 ± 5.00	0.049
SUA, mg/dL		4.42 ± 1.22	4.28 ± 1.15	4.27 ± 1.18	4.43 ± 1.23	4.68 ± 1.29	<0.001
HDL, mg/dL		51.59 ± 15.27	64.06 ± 15.38	54.92 ± 12.60	47.91 ± 10.49	39.45 ± 10.03	<0.001

Abbreviation: MCMI, modified cardiometabolic index; BMI, body mass index; WC, waist circumference; SBP, systolic blood pressure; DBP, diastolic blood pressure; FPG, fasting plasma glucose; HbA1c, glycated hemoglobin; TC, total cholesterol; TG, triglyceride; LDL‑C, low-density lipoprotein cholesterol; HDL-C, high-density lipoprotein cholesterol; SUA, serum uric acid; BUN, blood urea nitrogen; Scr, serum creatinine; GMS, glucose metabolism status; NGR, normal glucose regulation; CRP, C-reactive protein. The data are presented as mean ± SD or n (%).

Moreover, with increasing MCMI, the baseline proportions of participants who were urban residents, who were nonsmokers, who were nondrinkers, who were female, obese, hypertensive, diabetic, or dyslipidemic, who had a history of heart disease, and who were using antihypertensive, lipid-lowering, or cardiac medications also increased. Conversely, the proportions of rural residents and male participants decreased across MCMI quartiles.

### 3.2 The Prediction of New-Onset Stroke by MCMI

**Fig. 2.**
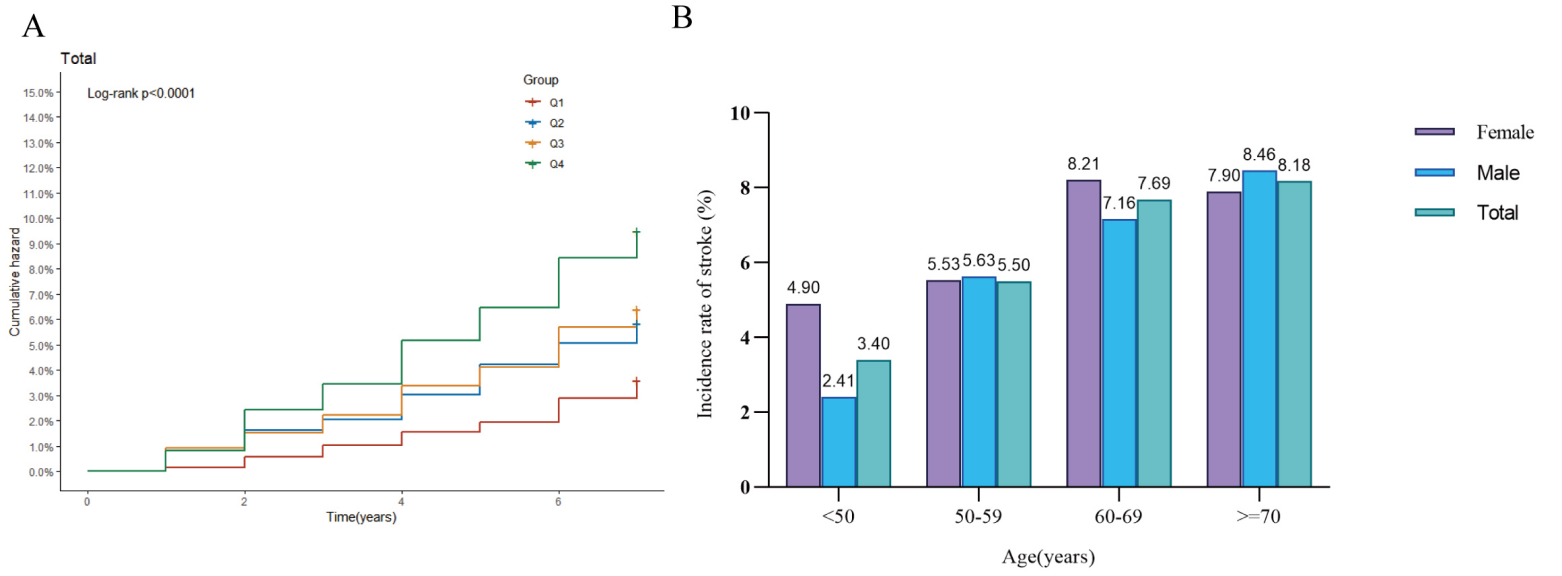
**Stroke incidence analysis**. (A) Kaplan–Meier curve showing the cumulative incidence of stroke across the four quartiles of MCMI; (B) Bar chart comparing stroke incidence by gender within different age groups.

During the 7-year follow-up period, 457 incident stroke cases (6.13%) were identified among the 7455 participants. The incidence of stroke increased progressively with higher baseline MCMI quartiles. Specifically, the number of new-onset stroke cases across quartiles Q1 to Q4 was 66 (3.54%), 106 (5.69%), 116 (6.23%), and 169 (9.07%), respectively. Kaplan‒Meier cumulative incidence curves indicated a stepwise increase in stroke events from Q1 to Q4 across all glycemic status subgroups (Fig. 2A). We next further compared stroke incidence by age and gender. When stratified by 10-year age groups, stroke incidence increased with age in males, while in females, it increased with age until approximately 70 years, after which it slightly decreased. Notably, among individuals aged <50 years and 60–69 years, the incidence of stroke was significantly higher in females than in age-matched males (Fig. 2B). To explore the relationship between the baseline MCMI and stroke risk, we developed three Cox proportional hazards regression models (Table 2). In Model 1 (unadjusted), a 1-unit increase in MCMI was associated with a 14% increase in stroke risk (HR = 1.14; 95% CI: 1.09–1.18). After adjusting for age and gender in Model 2, the association remained significant (HR = 1.15; 95% CI: 1.10–1.20). In the fully adjusted Model 3, which included additional covariates, each 1-unit increase in MCMI was associated with a 27% increase in stroke risk (HR = 1.27; 95% CI: 1.08–1.48). Furthermore, when the MCMI was divided into quartiles, multivariate-adjusted analyses revealed that, compared with the lowest quartile (Q1), the HRs for stroke were 1.57 (95% CI: 1.15–2.14) in Q2, 1.54 (95% CI: 1.12–2.13) in Q3, and 2.05 (95% CI: 1.45–2.89) in Q4. These findings suggest a graded increase in stroke risk of 57%, 54%, and 105% in Q2, Q3, and Q4, respectively, compared with Q1.

**Table 2.** **New stroke events based on MCMI in the three models**.

Characteristic		Model 1			Model 2			Model 3		
		HR	95% CI	*p*-value	HR	95% CI	*p*-value	HR	95% CI	*p*-value
MCMI		1.14	1.09–1.18	<0.001	1.15	1.10–1.20	<0.001	1.27	1.08–1.48	0.003
MCMI4										
	Q1	Ref.			Ref.			Ref.		
	Q2	1.63	1.20–2.22	0.002	1.67	1.23–2.28	0.001	1.57	1.15–2.14	0.005
	Q3	1.79	1.32–2.42	<0.001	1.88	1.39–2.56	<0.001	1.54	1.12–2.13	0.008
	Q4	2.65	1.99–3.52	<0.001	2.80	2.08–3.75	<0.001	2.05	1.45–2.89	<0.001

Abbreviation: MCMI, modified cardiometabolic index; HR, Hazard Ratio; CI, Confidence Interval. Model 1: Unadjusted. Model 2: Adjusted for age, gender, education level, marital status, place of residence, smoking, and drinking history. Model 3: Model 2 + adjusted for history of hypertension, dyslipidemia, heart disease, chronic kidney disease, use of antihypertensive drugs, use of lipid-lowering drugs, treatment with heart disease medications, body mass index, C-reactive protein, serum uric acid, blood urea nitrogen, serum creatinine.

### 3.3 Influence of Different Glucose Metabolism States on the Correlation Between MCMI and New-Onset Stroke

**Fig. 3.**
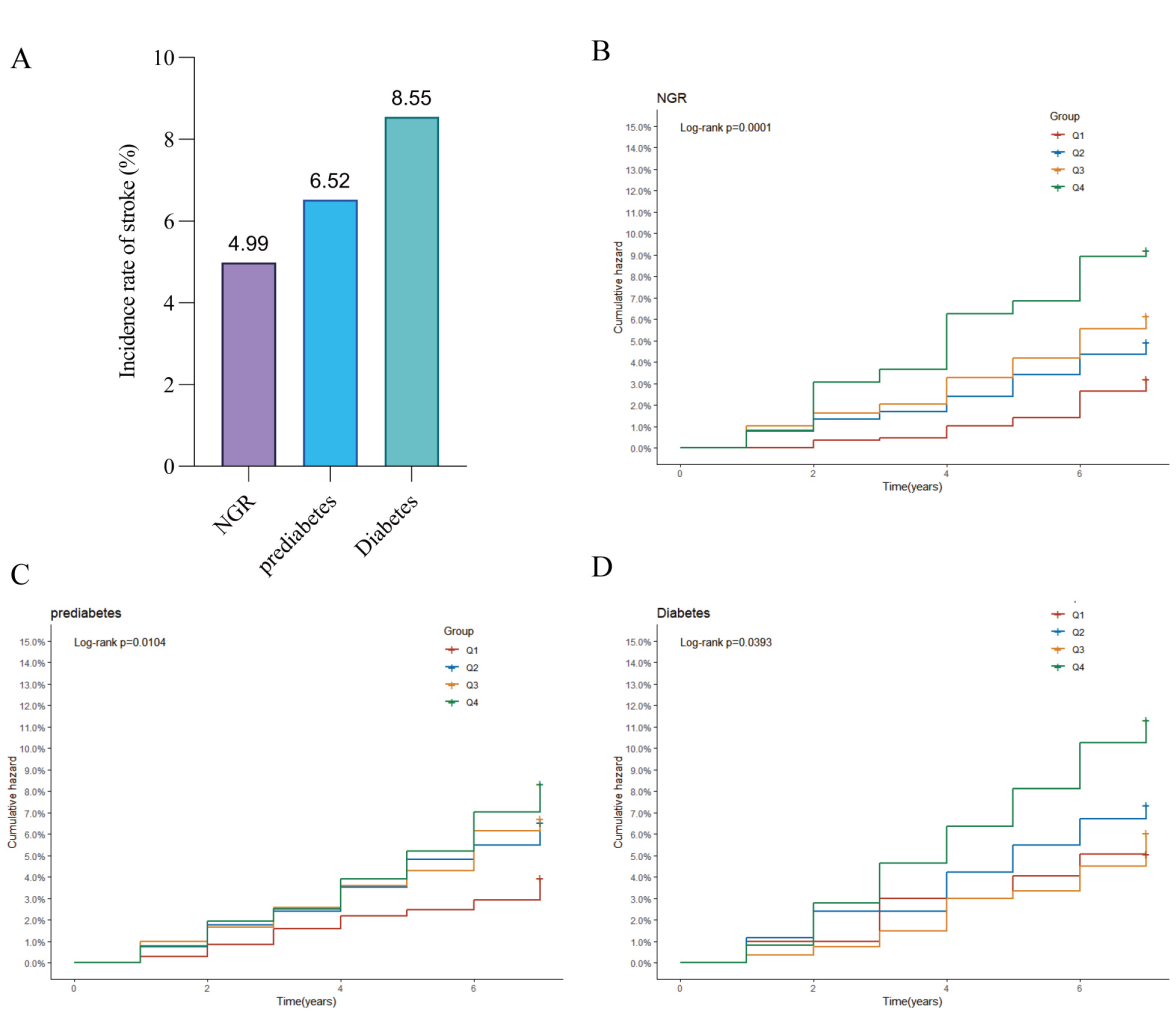
**Stroke incidence by blood glucose status**. (A) Bar chart of stroke incidence rates across different blood glucose status groups; (B) Kaplan–Meier curve of cumulative stroke incidence in participants with normal glucose regulation; (C) Kaplan–Meier curve of cumulative stroke incidence in pre-diabetic participants; (D) Kaplan–Meier curve of cumulative stroke incidence in diabetic participants.

During the follow-up period, 150 (4.99%) incident stroke cases occurred in the NGR group, 208 (6.32%) in the prediabetes and 99 (8.55%) in the diabetes group (Fig. 3A). Kaplan‒Meier cumulative incidence curve analysis revealed a progressive increase in stroke events from the Q1 to Q4 groups across all glycemic status categories, with statistically significant differences observed (Fig. 3B–D). The Cox proportional hazards regression results are presented in Table 3. Model 3 demonstrated a significant association between the MCMI and stroke risk in both NGR and prediabetic individuals. Although the NGR group showed no statistically significant association when MCMI was treated as a continuous variable, a significant increase in stroke risk with increasing MCMI was observed when the MCMI was analyzed as a categorical variable. These findings suggest a potentially nonlinear relationship between the MCMI and stroke occurrence in NGR individuals. For prediabetic individuals, each 1-unit increase in MCMI was associated with a 34% increase in stroke risk (HR =1.34, 95% CI: 1.03–1.75). However, no significant association was detected in diabetic individuals.

**Table 3.** **Association between MCMI and stroke incidence according to glucose regulation status**.

Characteristic		Model 1			Model 2			Model 3		
		HR	95% CI	*p*-value	HR	95% CI	*p*-value	HR	95% CI	*p*-value
NGR										
MCMI		1.22	1.06, 1.41	0.005	1.21	1.05, 1.40	0.01	1.34	0.93, 1.92	0.115
MCMI4										
	Q1	Ref.			Ref.			Ref.		
	Q2	1.54	0.98, 2.41	0.061	1.56	0.99, 2.46	0.054	1.51	0.95, 2.39	0.083
	Q3	1.92	1.22, 3.03	0.005	1.99	1.25, 3.17	0.004	1.85	1.12, 3.04	0.016
	Q4	2.88	1.78, 4.68	<0.001	2.94	1.78, 4.86	<0.001	2.59	1.43, 4.71	0.002
Prediabetes										
MCMI		1.1	1.03, 1.18	0.004	1.12	1.05, 1.19	0.001	1.34	1.03, 1.75	0.028
MCMI4										
	Q1	Ref.			Ref.			Ref.		
	Q2	1.66	1.04, 2.64	0.034	1.69	1.06, 2.70	0.028	1.64	1.02, 2.63	0.041
	Q3	1.7	1.08, 2.68	0.022	1.81	1.14, 2.87	0.012	1.51	0.93, 2.44	0.097
	Q4	2.11	1.35, 3.28	<0.001	2.3	1.45, 3.64	<0.001	1.7	1.00, 2.88	0.048
Diabetes										
MCMI		1.19	1.01, 1.39	0.035	1.18	1.00, 1.40	0.044	1.06	0.79, 1.42	0.689
MCMI4										
	Q1	Ref.			Ref.			Ref.		
	Q2	1.45	0.51, 4.11	0.487	1.39	0.49, 3.99	0.536	1.28	0.44, 3.69	0.648
	Q3	1.18	0.43, 3.23	0.744	1.14	0.41, 3.15	0.799	0.82	0.29, 2.32	0.709
	Q4	2.23	0.90, 5.52	0.085	2.18	0.86, 5.56	0.101	1.37	0.50, 3.72	0.539

Abbreviation: MCMI, modified cardiometabolic index; HR, Hazard Ratio; NGR, normal glucose regulation; CI, Confidence Interval. Model 1: Unadjusted. Model 2: Adjusted for age, gender, education level, marital status, place of residence, smoking, and drinking history. Model 3: Model 2 + adjusted for history of hypertension, dyslipidemia, heart disease, chronic kidney disease, use of antihypertensive drugs, use of lipid-lowering drugs, treatment with heart disease medications, body mass index, C-reactive protein, serum uric acid, blood urea nitrogen, serum creatinine.

**Fig. 4.**
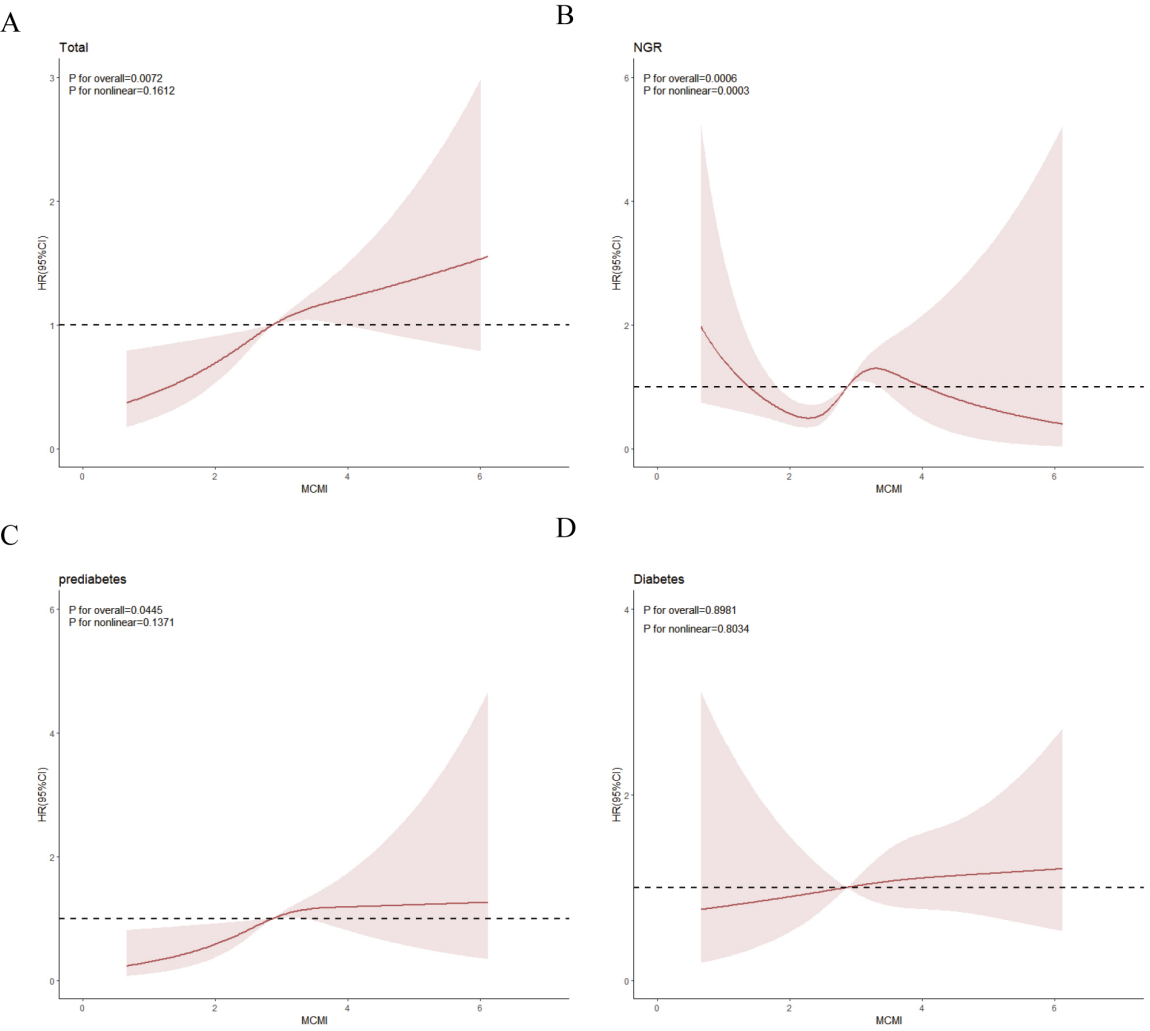
**The association between the MCMI index and the risk of stroke as analyzed by RCS**. All participants (A); NGR participants (B); participants with prediabetes (C); participants with diabetes (D). RCS, restricted cubic spline.

RCS analysis indicated an overall significant linear relationship between MCMI and stroke events (Fig. 4A). A significant nonlinear association was observed in the NGR group (Fig. 4B), while prediabetic individuals showed a significant linear relationship (Fig. 4C). Conversely, no significant dose-response correlation was detected between the MCMI and stroke risk in diabetic patients (Fig. 4D). Given the nonlinear relationship between the MCMI and stroke events in the NGR group, we identified an inflection point for the MCMI at 1.904 using threshold analysis. Subsequent two-piece Cox proportional hazards regression modeling was used to determine the HRs and CIs on either side of this threshold. As shown in Table 4, the HR was 0.29 (95% CI: 0.12–0.67) before the inflection point and 1.85 (95% CI: 1.24–2.76) thereafter.

**Table 4.** **Two linear regression models in the normal glucose regulation group**.

Outcome: incident stroke		HR (95% CI)	*p* value
Fitting model by standard linear regression		1.34 (0.93–1.92)	0.115
Fitting model by two-piecewise linear regression			
Inflection point		1.904	
	<1.904	0.29 (0.12–0.67)	0.004
	>1.904	1.85 (1.24–2.76)	0.003
*p* for the likelihood ratio test		0.002	

Model: The model takes into account age, gender, educational level, marital status, place of residence, smoking, drinking, history of hypertension, abnormal blood lipid levels, history of heart disease, chronic kidney disease, use of antihypertensive drugs, use of lipid-lowering drugs, heart disease treatment, body mass index, C-reactive protein, serum uric acid, blood urea nitrogen, serum creatinine.

### 3.4 Subgroup Analysis

**Fig. 5.**
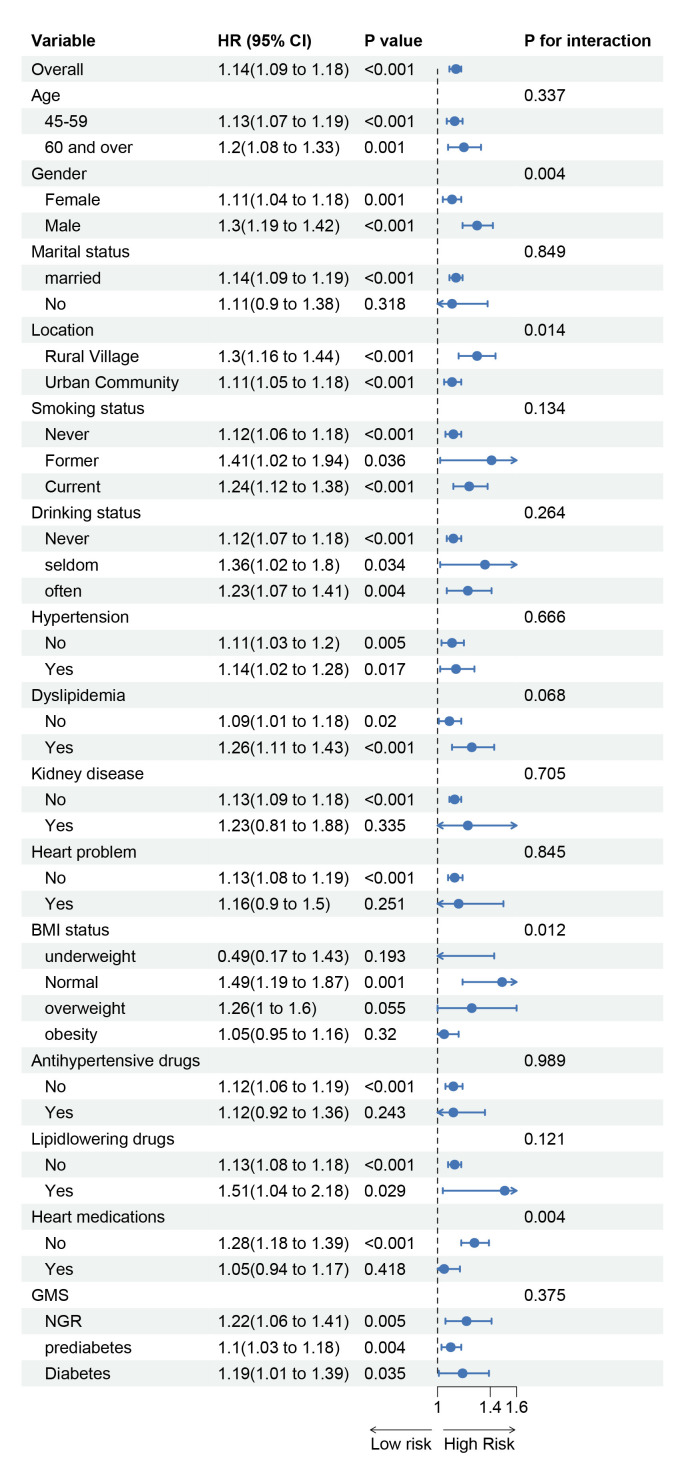
**Subgroup and interaction analysis of the association between MCMI and stroke risk**.

**Fig. 6.**
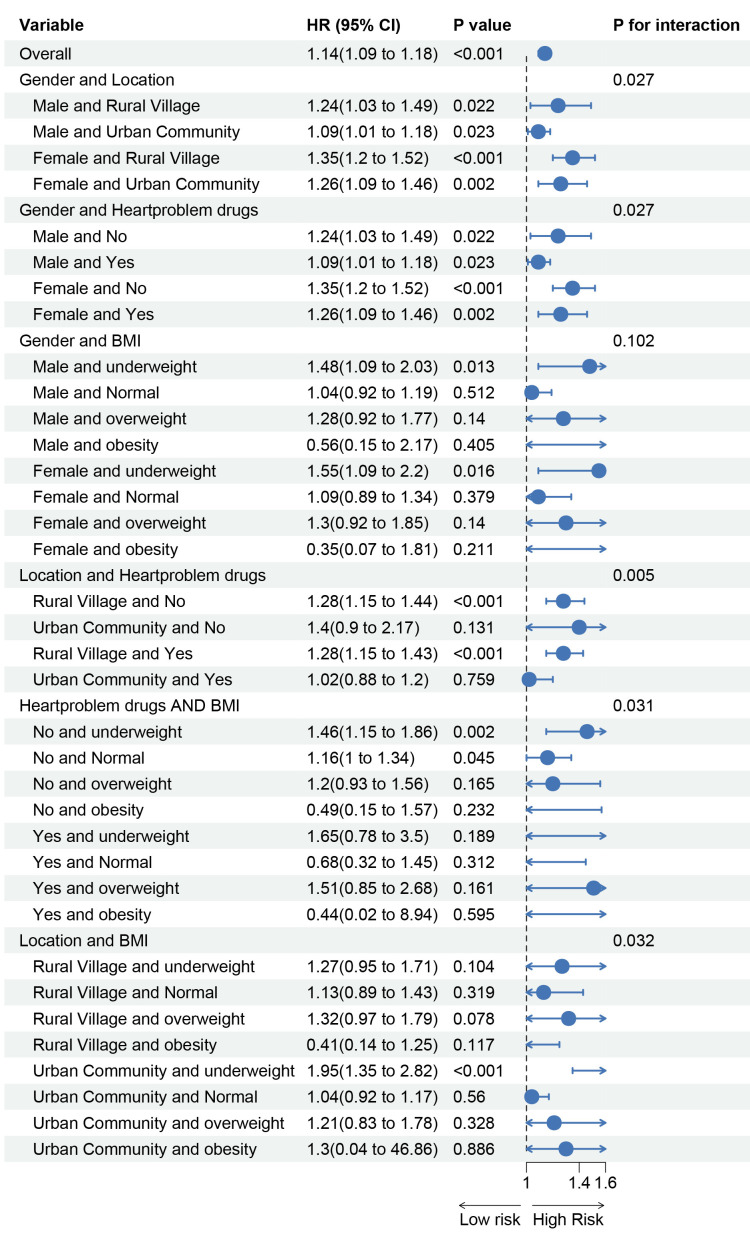
**Reanalysis based on the meaningful subgroups shown in Fig. 5**.

Subgroup analyses were performed based on gender, age, education level, residence (urban/rural), marital status, smoking status, alcohol consumption, BMI, relevant medical history, and medication use. The results (Fig. 5) indicate that the association between MCMI and incident stroke varied by gender, residence, BMI, and cardiac medication use. This suggests that male gender, rural residence, normal weight, and no use of cardiac medications were associated with a relatively higher risk of MCMI-related stroke. We further explored the combined effects of these high-risk factors on the association between MCMI and stroke. The results revealed that females living in rural areas, those who did not receive cardiac medication treatment, and individuals who were underweight in rural areas had a relatively higher risk of developing MCMI-related stroke (Fig. 6).

### 3.5 Sensitivity Analysis and Diagnostic Performance

To assess the robustness of our findings, we conducted multiple sensitivity analyses. First, after all missing data were excluded, the results remained largely unchanged (**Supplementary Tables 3,4**; **Supplementary Figs. 1–3**). Second, when we removed all deceased participants and reanalyzed the data, no substantial changes were observed (**Supplementary Tables 5,6**; **Supplementary Figs. 4–6**). Third, the results remained stable when a logistic regression model was used (**Supplementary Tables 7,8**; **Supplementary Fig. 7**). Additionally, the E value for MCMI was calculated based on Model 3, yielding an E value of 1.86. This suggests that a relatively strong unmeasured confounder would be needed to fully explain the observed HRs. The diagnostic performance of the MCMI, CMI, TyG index, and WHtR for the identification of new strokes is shown in **Supplementary Fig. 8**. The MCMI has the highest AUC for identifying strokes, with a value of 0.599, which is superior to those of the CMI (AUC = 0.578), TyG index (AUC = 0.576), and WHtR (AUC = 0.589). **Supplementary Tables. 9–12** contain additional data provided in response to reviewer comments.

## 4. Discussion

This study revealed a positive correlation between the MCMI and stroke risk. Notably, among participants with different glycemic statuses, the MCMI showed a significant nonlinear association with stroke risk in those with NGR. When the MCMI exceeded 1.904, the risk increased significantly. Higher MCMI levels were associated with increased stroke risk in prediabetic individuals. To our knowledge, this is the first study to investigate the relationship between the MCMI and stroke incidence among middle-aged and elderly Chinese individuals across different glucose metabolism states, providing new insights for stroke prevention, early diagnosis, and control.

The MCMI was developed by Guo *et al*. [20] and was initially used to predict NAFLD and liver fibrosis incidence. This index combines the CMI, TyG, and WHtR indices, and existing research has clearly demonstrated associations between these three indices and stroke risk. A meta-analysis revealed that the CMI, by integrating multiple cardiovascular and metabolic factors, is strongly correlated with increased stroke risk and can serve as a comprehensive predictor of stroke risk [29]. The TyG index is an independent risk factor for stroke and can predict stroke onset, recurrence, and mortality risk [17, 30]. Additionally, compared with other obesity indicators, such as BMI and WC, the WHtR is significantly positively correlated with stroke risk and has better predictive value [31, 32]. Therefore, we initially hypothesized that the MCMI might be related to stroke, and our study confirmed an association between the MCMI and stroke, supporting the hypothesis that an elevated MCMI is positively correlated with stroke risk. This comprehensive approach could help clinicians identify high-risk patients for stroke, enabling accurate risk assessment and targeted interventions to reduce stroke incidence.

Although the underlying mechanisms linking the MCMI to stroke are not fully understood, the MCMI is influenced primarily by fasting glucose levels, lipid levels, and the waist-to-height ratio. A higher MCMI may reflect states of hyperglycemia, dyslipidemia, or visceral fat accumulation. Hyperglycemia, dyslipidemia, and elevated WHtR can independently increase stroke risk [33, 34, 35]. The potential mechanisms through which chronic hyperglycemia contributes to stroke include vascular endothelial dysfunction, the promotion of atherosclerotic plaque formation, and increased cholesterol levels through pathways such as the polyol pathway, protein kinase C activation, and advanced glycation end product accumulation [36, 37]. Additionally, hyperglycemia increases blood viscosity, reduces erythrocyte deformability, and promotes platelet aggregation and thrombosis [38]. Similarly, dyslipidemia increases ischemic stroke risk by promoting atherosclerosis, activating the coagulation system, increasing blood viscosity, and inhibiting the fibrinolytic system, leading to thrombosis and hemodynamic abnormalities [39, 40]. The waist-to-height ratio, as a core indicator of visceral obesity, directly reflects excessive abdominal fat accumulation, and visceral obesity has been identified as a key risk factor for stroke [41, 42]. Therefore, this study highlights the clinical importance of maintaining lower MCMI levels.

Analysis stratified by glycemic status revealed that the MCMI was significantly associated with stroke risk in NGR and prediabetic individuals, but no such association was observed in diabetic individuals. The lack of a significant association between the MCMI and stroke in diabetic patients may be attributed to several factors. In patients with diabetes, stroke risk may be primarily caused by mechanisms directly caused by hyperglycemia, such as vascular endothelial damage and coagulation abnormalities, rather than by dyslipidemia or visceral fat metabolism [43]. Diabetes is a strong risk factor for stroke, increasing ischemic stroke risk by 2- to 4-fold [44]. Thus, diabetes may overshadow the role of the MCMI as the dominant risk factor. Additionally, patients with diabetes often have other complications, such as nephropathy and neuropathy, which may contribute more significantly to stroke risk, diluting the impact of the MCMI [45, 46, 47]. Most importantly, the RCS results revealed that while stroke risk generally increased with an increasing MCMI, the association plateaued at very high MCMI levels. Compared with NGR and prediabetic individuals, diabetic individuals typically have MCMI levels in the higher range, where glucose, lipids, and visceral fat are already at stable high levels, reducing the sensitivity of the MCMI in predicting stroke risk [48]. These results emphasize the potential of the MCMI as an early marker for stroke and highlight the importance of managing MCMI levels based on glucose metabolism status. Specifically, the MCMI should be maintained below 1.904 in individuals with NGR, while prediabetic individuals should aim to lower MCMI levels to reduce stroke risk.

Subgroup analysis revealed significant relationships between the MCMI and stroke, with higher MCMI-related stroke risk observed in males, rural residents, normal-weight individuals, and those who did not use cardiac medications. These findings align with previous studies. In terms of gender differences, the global stroke incidence is 33% greater in males than in females, and abdominal obesity is more common in males, significantly increasing stroke risk even among individuals with a normal weight [49, 50]. With respect to rural–urban disparities, stroke prevalence is higher in rural China [51]. With respect to medication differences, cardiac patients who do not use anticoagulants/antiplatelet drugs have a significantly greater risk of stroke [52]. The higher MCMI-related stroke risk in normal-weight individuals may be due to visceral fat accumulation in some normal-BMI individuals, which increases stroke risk through metabolic disturbances. In contrast, in obese individuals, obesity itself and associated metabolic abnormalities may dominate stroke risk, masking the independent effects of the MCMI.

## 5. Limitations

Our findings may significantly influence future randomized trials focused on stroke and atherosclerotic cardiovascular disease prevention, potentially benefiting from selectively recruiting high-risk individuals and targeting MCMI reduction. However, several limitations should be acknowledged. First, while the CHARLS dataset provides representative data for Chinese middle-aged and elderly people, it may not fully reflect broader population diversity. Second, residual confounding is possible due to the observational design. Although known covariates were adjusted for, unmeasured variables such as dietary intake, physical activity, and medication adherence were not included, potentially leading to over- or underestimation of the true association. Third, using self-reported physician-diagnosed stroke may have introduced recall or reporting bias, particularly in individuals with limited health literacy, possibly diluting association strength and subgroup analysis accuracy. Finally, the study did not account for changes in glycemic status or MCMI levels over time. Future research should consider these dynamic effects for a more comprehensive understanding.

## 6. Conclusion

In this study, we demonstrated that the MCMI serves as a valuable predictor of stroke risk in middle-aged and elderly Chinese individuals. Our findings revealed significant associations between the MCMI and stroke incidence among individuals with NGR and those with prediabetes, across different glycemic statuses. These results underscore the necessity of developing tailored risk management strategies based on individual glucose metabolic profiles. Optimizing diet structure, increasing moderate-intensity aerobic exercise, and controlling body weight are recommended to comprehensively improve the metabolic health of the heart and improve the MCMI value. Particularly in resource-limited settings where access to advanced biomarker measurement or imaging technologies may be constrained, incorporating MCMI measurements into routine clinical practice could significantly enhance early identification of individuals at high risk for stroke. The simplicity and cost-effectiveness of MCMI calculation make it particularly suitable for widespread clinical application in such environments.

## Availability of Data and Materials

All relevant data are described within the paper. Deidentified data can be requested. Data can be requested by all interested researchers, who can be contacted via the corresponding author.

## Supplementary Material

Supplementary material associated with this article can be found, in the online version, at https://doi.org/10.31083/RCM45989.
