# Supplementary material for: Associations Between the Modified Cardiometabolic Index and Stroke in Patients With Different Glucose Metabolism Statuses: Evidence From a Nationally Representative Survey
Source: Rev Cardiovasc Med. 2026 Mar 19;27(3):45989. doi: 10.31083/RCM45989 (PMC13036548; doi:10.31083/RCM45989)
Supplement: Supplementary file 1 [file 2153-8174-27-3-45989-s1.zip › Supplementary Material.docx]

**Supplement materials**

Supplementary Table 1. Number of missing covariates.

| Variable | Count |
| --- | --- |
| smoking | 14 |
| drinking | 385 |
| heartproblem | 32 |
| kidneydisease | 41 |
| Antihypertensive drugs | 41 |
| Heart problem medications | 32 |
| Lipidlowering drugs | 161 |
| bmi | 14 |
| weight | 14 |
| BUN | 1 |
| Scr | 4 |
| Hypertension | 21 |
| Dyslipidemia | 1 |

Supplementary Table 2. Variance inflation factor table.

| Variable | VIF |
| --- | --- |
| MCMI | 9.769369319 |
| age | 1.619102028 |
| Gender | 3.00341413 |
| Education | 1.457665292 |
| Marital status | 1.130208366 |
| Location | 1.100219407 |
| Drinking status | 1.428939545 |
| Smoking status | 2.062141609 |
| Heartproblem | 1.655612124 |
| Hypertension | 2.526088211 |
| Dyslipidemia | 1.649670857 |
| Antihypertensive drugs | 1.636921308 |
| Heart problem medications | 1.14503507 |
| Lipidlowering drugs | 1.665212807 |
| Kidney disease | 1.020476448 |
| SUA | 1.555535769 |
| CRP | 1.013021151 |
| BUN | 1.152405876 |
| Scr | 1.732193671 |
| bmi | 3.193309222 |
| TC | 7.094102558 |
| TG | 5.603478694 |
| LDL | 6.773102695 |

Supplementary Table 3. Stroke hazard ratio based on MCMI in the three models (after removing all data with missing covariates).

|  | Model 1 | | |  | Model 2 | | |  | Model 3 | | |
| --- | --- | --- | --- | --- | --- | --- | --- | --- | --- | --- | --- |
| Characteristic | HR | 95% CI | p-value |  | HR | 95% CI | p-value |  | HR | 95% CI | p-value |
| MCMI | 1.13 | 1.08, 1.18 | <0.001 |  | 1.15 | 1.10, 1.20 | <0.001 |  | 1.28 | 1.08, 1.51 | 0.004 |
| MCMI4 |  |  |  |  |  |  |  |  |  |  |  |
| Q1 | Ref. | | |  | Ref. | | |  | Ref. | | |
| Q2 | 1.55 | 1.13, 2.14 | 0.007 |  | 1.59 | 1.15, 2.19 | 0.005 |  | 1.49 | 1.08, 2.07 | 0.016 |
| Q3 | 1.70 | 1.24, 2.33 | <0.001 |  | 1.78 | 1.29, 2.45 | <0.001 |  | 1.45 | 1.04, 2.04 | 0.029 |
| Q4 | 2.51 | 1.87, 3.38 | <0.001 |  | 2.64 | 1.94, 3.58 | <0.001 |  | 1.93 | 1.35, 2.78 | <0.001 |

Abbreviation: MCMI: modified cardiometabolic index; HR: Hazard Ratio; CI: Confidence Interval.

Model 1: Unadjusted.

Model 2: Adjusted for age, gender, education level, marital status, place of residence, smoking and drinking history.

Model 3: Model 2 + adjusted for history of hypertension, dyslipidemia, heart disease, chronic kidney disease, use of antihypertensive drugs, use of lipid-lowering drugs, treatment with heart disease medications, body mass index, C-reactive protein, serum uric acid, blood urea nitrogen, serum Creatinine.

Supplementary Table 4. The association between the MCMI under glucose regulation status and the incidence of stroke (after removing all data with missing covariates).

|  | Model 1 | | |  | Model 2 | | |  | Model 3 | | |
| --- | --- | --- | --- | --- | --- | --- | --- | --- | --- | --- | --- |
| Characteristic | HR | 95% CI | p-value |  | HR | 95% CI | p-value |  | HR | 95% CI | p-value |
| NGR | | | | | | | | | | | |
| MCMI | 1.21 | 1.05, 1.41 | 0.011 |  | 1.21 | 1.03, 1.41 | 0.017 |  | 1.32 | 0.91, 1.93 | 0.147 |
| MCMI4 |  |  |  |  |  |  |  |  |  |  |  |
| Q1 | Ref. | | |  | Ref. | | |  | Ref. | | |
| Q2 | 1.43 | 0.89, 2.27 | 0.136 |  | 1.45 | 0.91, 2.33 | 0.120 |  | 1.43 | 0.88, 2.30 | 0.147 |
| Q3 | 1.83 | 1.15, 2.91 | 0.011 |  | 1.88 | 1.17, 3.03 | 0.010 |  | 1.78 | 1.06, 2.97 | 0.028 |
| Q4 | 2.71 | 1.64, 4.46 | <0.001 |  | 2.75 | 1.64, 4.61 | <0.001 |  | 2.45 | 1.32, 4.53 | 0.005 |
| Prediabetes | | | | | | | | | | | |
| MCMI | 1.10 | 1.02, 1.18 | 0.009 |  | 1.11 | 1.04, 1.20 | 0.003 |  | 1.35 | 1.02, 1.79 | 0.038 |
| MCMI4 |  |  |  |  |  |  |  |  |  |  |  |
| Q1 | Ref. | | |  | Ref. | | |  | Ref. | | |
| Q2 | 1.54 | 0.94, 2.51 | 0.085 |  | 1.55 | 0.95, 2.53 | 0.081 |  | 1.53 | 0.93, 2.51 | 0.095 |
| Q3 | 1.62 | 1.01, 2.61 | 0.047 |  | 1.70 | 1.05, 2.76 | 0.031 |  | 1.41 | 0.85, 2.35 | 0.183 |
| Q4 | 1.97 | 1.24, 3.14 | 0.004 |  | 2.10 | 1.30, 3.41 | 0.003 |  | 1.57 | 0.90, 2.75 | 0.112 |
| Diabetes | | | | | | | | | | | |
| MCMI | 1.19 | 1.01, 1.40 | 0.035 |  | 1.18 | 1.01, 1.39 | 0.041 |  | 1.09 | 0.80, 1.49 | 0.569 |
| MCMI4 |  |  |  |  |  |  |  |  |  |  |  |
| Q1 | Ref. | | | | Ref. | | |  | Ref. | | |
| Q2 | 1.81 | 0.58, 5.62 | 0.304 |  | 1.75 | 0.56, 5.47 | 0.336 |  | 1.64 | 0.52, 5.16 | 0.397 |
| Q3 | 1.26 | 0.41, 3.82 | 0.687 |  | 1.22 | 0.40, 3.78 | 0.724 |  | 0.86 | 0.27, 2.69 | 0.791 |
| Q4 | 2.50 | 0.91, 6.89 | 0.075 |  | 2.52 | 0.89, 7.12 | 0.081 |  | 1.52 | 0.51, 4.52 | 0.453 |

Abbreviation: MCMI: modified cardiometabolic index; HR: Hazard Ratio; CI: Confidence Interval.

Model 1: Unadjusted.

Model 2: Adjusted for age, gender, education level, marital status, place of residence, smoking and drinking history.

Model 3: Model 2 + adjusted for history of hypertension, dyslipidemia, heart disease, chronic kidney disease, use of antihypertensive drugs, use of lipid-lowering drugs, treatment with heart disease medications, body mass index, C-reactive protein, serum uric acid, blood urea nitrogen, serum Creatinine.

Supplementary Table 5. Stroke HR based on MCMI in the three models (after excluding data of the deceased population).

|  | Model 1 | | |  | Model 2 | | |  | Model 3 | | |
| --- | --- | --- | --- | --- | --- | --- | --- | --- | --- | --- | --- |
| Characteristic | HR | 95% CI | p-value |  | HR | 95% CI | p-value |  | HR | 95% CI | p-value |
| MCMI | 1.14 | 1.09, 1.18 | <0.001 |  | 1.15 | 1.10, 1.20 | <0.001 |  | 1.26 | 1.08, 1.48 | 0.003 |
| MCMI4 |  |  |  |  |  |  |  |  |  |  |  |
| Q1 | Ref. | | |  | Ref. | | |  | Ref. | | |
| Q2 | 1.64 | 1.20, 2.23 | 0.002 |  | 1.68 | 1.23, 2.30 | 0.001 |  | 1.57 | 1.15, 2.15 | 0.005 |
| Q3 | 1.77 | 1.30, 2.40 | <0.001 |  | 1.86 | 1.37, 2.54 | <0.001 |  | 1.52 | 1.10, 2.10 | 0.012 |
| Q4 | 2.69 | 2.02, 3.58 | <0.001 |  | 2.84 | 2.12, 3.82 | <0.001 |  | 2.05 | 1.45, 2.91 | <0.001 |

Abbreviation: MCMI: modified cardiometabolic index; HR: Hazard Ratio; CI: Confidence Interval.

Model 1: Unadjusted.

Model 2: Adjusted for age, gender, education level, marital status, place of residence, smoking and drinking history.

Model 3: Model 2 + adjusted for history of hypertension, dyslipidemia, heart disease, chronic kidney disease, use of antihypertensive drugs, use of lipid-lowering drugs, treatment with heart disease medications, body mass index, C-reactive protein, serum uric acid, blood urea nitrogen, serum Creatinine.

Supplementary Table 6. The association between the MCMI reflecting the glucose regulation status and the incidence of stroke (after excluding the data of the deceased population).

|  | Model 1 | | |  | Model 2 | | |  | Model 3 | | |
| --- | --- | --- | --- | --- | --- | --- | --- | --- | --- | --- | --- |
| Characteristic | HR | 95% CI | p-value |  | HR | 95% CI | p-value |  | HR | 95% CI | p-value |
| NGR | | | | | | | | | | | |
| MCMI | 1.22 | 1.06, 1.41 | 0.006 |  | 1.21 | 1.05, 1.41 | 0.010 |  | 1.31 | 0.91, 1.88 | 0.147 |
| MCMI4 |  |  |  |  |  |  |  |  |  |  |  |
| Q1 | Ref. | | |  | Ref. | | |  | Ref. | | |
| Q2 | 1.51 | 0.96, 2.37 | 0.074 |  | 1.53 | 0.97, 2.42 | 0.066 |  | 1.47 | 0.92, 2.34 | 0.104 |
| Q3 | 1.88 | 1.19, 2.96 | 0.007 |  | 1.95 | 1.22, 3.11 | 0.005 |  | 1.79 | 1.09, 2.96 | 0.023 |
| Q4 | 2.88 | 1.78, 4.67 | <0.001 |  | 2.95 | 1.79, 4.87 | <0.001 |  | 2.51 | 1.38, 4.57 | 0.003 |
| prediabetes | | | | | | | | | | | |
| MCMI | 1.10 | 1.03, 1.18 | 0.003 |  | 1.12 | 1.05, 1.19 | <0.001 |  | 1.35 | 1.04, 1.76 | 0.024 |
| MCMI4 |  |  |  |  |  |  |  |  |  |  |  |
| Q1 | Ref. | | |  | Ref. | | |  | Ref. | | |
| Q2 | 1.71 | 1.07, 2.74 | 0.026 |  | 1.75 | 1.09, 2.81 | 0.021 |  | 1.69 | 1.05, 2.73 | 0.032 |
| Q3 | 1.73 | 1.09, 2.74 | 0.021 |  | 1.83 | 1.15, 2.93 | 0.011 |  | 1.53 | 0.93, 2.49 | 0.091 |
| Q4 | 2.19 | 1.40, 3.43 | <0.001 |  | 2.39 | 1.50, 3.80 | <0.001 |  | 1.76 | 1.03, 3.00 | 0.037 |
| Diabetes | | | | | | | | | | | |
| MCMI | 1.19 | 1.02, 1.39 | 0.031 |  | 1.19 | 1.01, 1.40 | 0.041 |  | 1.07 | 0.80, 1.43 | 0.653 |
| MCMI4 |  |  |  |  |  |  |  |  |  |  |  |
| Q1 | Ref. | | | | Ref. | | |  | Ref. | | |
| Q2 | 1.45 | 0.51, 4.11 | 0.487 |  | 1.41 | 0.49, 4.03 | 0.523 |  | 1.28 | 0.44, 3.69 | 0.646 |
| Q3 | 1.12 | 0.41, 3.10 | 0.820 |  | 1.10 | 0.40, 3.07 | 0.852 |  | 0.79 | 0.28, 2.24 | 0.652 |
| Q4 | 2.24 | 0.90, 5.55 | 0.083 |  | 2.21 | 0.87, 5.63 | 0.096 |  | 1.37 | 0.50, 3.73 | 0.535 |

Abbreviation: MCMI: modified cardiometabolic index; HR: Hazard Ratio; CI: Confidence Interval.

Model 1: Unadjusted.

Model 2: Adjusted for age, gender, education level, marital status, place of residence, smoking and drinking history.

Model 3: Model 2 + adjusted for history of hypertension, dyslipidemia, heart disease, chronic kidney disease, use of antihypertensive drugs, use of lipid-lowering drugs, treatment with heart disease medications, body mass index, C-reactive protein, serum uric acid, blood urea nitrogen, serum Creatinine.

Supplementary Table 7. Stroke HR based on MCMI in the three models (Logistic Regression).

|  | Model 1 | | |  | Model 2 | | |  | Model 3 | | |
| --- | --- | --- | --- | --- | --- | --- | --- | --- | --- | --- | --- |
| Characteristic | OR | 95% CI | p-value |  | OR | 95% CI | p-value |  | OR | 95% CI | p-value |
| MCMI | 1.27 | 1.15, 1.41 | <0.001 |  | 1.27 | 1.15, 1.42 | <0.001 |  | 1.29 | 1.09, 1.52 | 0.003 |
| MCMI4 |  |  |  |  |  |  |  |  |  |  |  |
| Q1 | Ref. | | |  | Ref. | | |  | Ref. | | |
| Q2 | 1.64 | 1.20, 2.26 | 0.002 |  | 1.69 | 1.23, 2.33 | 0.001 |  | 1.58 | 1.15, 2.19 | 0.005 |
| Q3 | 1.81 | 1.33, 2.48 | <0.001 |  | 1.91 | 1.40, 2.63 | <0.001 |  | 1.57 | 1.13, 2.20 | 0.008 |
| Q4 | 2.72 | 2.04, 3.66 | <0.001 |  | 2.88 | 2.14, 3.92 | <0.001 |  | 2.10 | 1.47, 3.02 | <0.001 |

Abbreviation: MCMI: modified cardiometabolic index; HR: Hazard Ratio; CI: Confidence Interval.

Model 1: Unadjusted.

Model 2: Adjusted for age, gender, education level, marital status, place of residence, smoking and drinking history.

Model 3: Model 2 + adjusted for history of hypertension, dyslipidemia, heart disease, chronic kidney disease, use of antihypertensive drugs, use of lipid-lowering drugs, treatment with heart disease medications, body mass index, C-reactive protein, serum uric acid, blood urea nitrogen, serum Creatinine.

Supplementary Table 8. The association (Logistic Regression) between the MCMI based on glucose regulation status and the incidence of stroke.

|  | Model 1 | | |  | Model 2 | | |  | Model 3 | | |
| --- | --- | --- | --- | --- | --- | --- | --- | --- | --- | --- | --- |
| Characteristic | OR | 95% CI | p-value |  | OR | 95% CI | p-value |  | OR | 95% CI | p-value |
| NGR | | | | | | | | | | | |
| MCMI | 1.26 | 1.04, 1.50 | 0.010 |  | 1.25 | 1.03, 1.50 | 0.016 |  | 1.34 | 0.93, 1.96 | 0.128 |
| MCMI4 |  |  |  |  |  |  |  |  |  |  |  |
| Q1 | Ref. | | |  | Ref. | | |  | Ref. | | |
| Q2 | 1.54 | 0.98, 2.46 | 0.064 |  | 1.58 | 0.99, 2.52 | 0.055 |  | 1.52 | 0.95, 2.46 | 0.084 |
| Q3 | 1.94 | 1.22, 3.10 | 0.005 |  | 2.03 | 1.26, 3.29 | 0.004 |  | 1.89 | 1.13, 3.19 | 0.015 |
| Q4 | 2.94 | 1.78, 4.84 | <0.001 |  | 3.00 | 1.78, 5.05 | <0.001 |  | 2.65 | 1.42, 4.95 | 0.002 |
| prediabetes | | | | | | | | | | | |
| MCMI | 1.16 | 1.03, 1.37 | 0.033 |  | 1.19 | 1.04, 1.42 | 0.022 |  | 1.37 | 1.04, 1.82 | 0.027 |
| MCMI4 |  |  |  |  |  |  |  |  |  |  |  |
| Q1 | Ref. | | |  | Ref. | | |  | Ref. | | |
| Q2 | 1.67 | 1.05, 2.73 | 0.035 |  | 1.71 | 1.07, 2.81 | 0.028 |  | 1.66 | 1.03, 2.74 | 0.043 |
| Q3 | 1.72 | 1.09, 2.78 | 0.022 |  | 1.84 | 1.15, 2.99 | 0.012 |  | 1.52 | 0.93, 2.54 | 0.100 |
| Q4 | 2.16 | 1.39, 3.46 | <0.001 |  | 2.36 | 1.49, 3.84 | <0.001 |  | 1.73 | 1.01, 3.02 | 0.050 |
| Diabetes | | | | | | | | | | | |
| MCMI | 1.22 | 1.00, 1.49 | 0.045 |  | 1.22 | 0.99, 1.50 | 0.051 |  | 1.08 | 0.79, 1.47 | 0.640 |
| MCMI4 |  |  |  |  |  |  |  |  |  |  |  |
| Q1 | Ref. | | | | Ref. | | |  | Ref. | | |
| Q2 | 1.47 | 0.53, 4.73 | 0.484 |  | 1.39 | 0.49, 4.52 | 0.555 |  | 1.24 | 0.43, 4.11 | 0.700 |
| Q3 | 1.20 | 0.46, 3.75 | 0.729 |  | 1.15 | 0.43, 3.65 | 0.789 |  | 0.82 | 0.30, 2.67 | 0.726 |
| Q4 | 2.30 | 0.99, 6.71 | 0.080 |  | 2.25 | 0.94, 6.70 | 0.098 |  | 1.38 | 0.53, 4.38 | 0.539 |

Abbreviation: MCMI: modified cardiometabolic index; HR: Hazard Ratio; CI: Confidence Interval.

Model 1: Unadjusted.

Model 2: Adjusted for age, gender, education level, marital status, place of residence, smoking and drinking history.

Model 3: Model 2 + adjusted for history of hypertension, dyslipidemia, heart disease, chronic kidney disease, use of antihypertensive drugs, use of lipid-lowering drugs, treatment with heart disease medications, body mass index, C-reactive protein, serum uric acid, blood urea nitrogen, serum creatinine.

Supplementary Table 9. Study inclusion/exclusion criteria baseline characteristics.

|  | level | Overall | Exclude | Include | p |
| --- | --- | --- | --- | --- | --- |
| n |  | 17708 | 10253 | 7455 |  |
| age(mean SD) |  | 58.50(10.17) | 58.27(10.91) | 58.80(9.08) | 0.001 |
| Age(%) | 45-59 | 10243(58.42) | 5986(59.40) | 4257(57.10) | 0.002 |
|  | 60andover | 7290(41.58) | 4092(40.60) | 3198(42.90) |  |
| Gender(%) | Female | 9228(52.12) | 5192(50.65) | 4036(54.14) | <0.001 |
|  | Male | 8478(47.88) | 5059(49.35) | 3419(45.86) |  |
| BMI(%) | <24 | 8144(59.75) | 3750(60.58) | 4394(59.05) | 0.072 |
|  | >=24 | 5487(40.25) | 2440(39.42) | 3047(40.95) |  |
| Lipidloweringdrugs(%) | No | 16336(94.97) | 9399(94.87) | 6937(95.11) | 0.512 |
|  | Yes | 865(5.03) | 508(5.13) | 357(4.89) |  |

Supplementary Table 10. Competitiveness Risk Model VIF.

| Variable | VIF |
| --- | --- |
| MCMI | 2.00972247 |
| age | 1.373887275 |
| Education | 1.350904332 |
| maritalstatus | 1.119951621 |
| Location | 1.090919359 |
| drinking | 1.29611139 |
| smoking | 1.422053502 |
| heartproblem | 1.651405451 |
| Hypertension | 1.585213989 |
| Dyslipidemia | 1.38319469 |
| Antihypertensivedrugs | 1.611755037 |
| Lipidloweringdrugs | 1.140584647 |
| heartproblemdrugs | 1.658442861 |
| kidneydisease | 1.018336738 |
| SUA | 1.500063132 |
| CRP | 1.010018024 |
| BUN | 1.138912166 |
| Scr | 1.595672071 |
| BMI | 1.540526953 |

The Proportional Hazards test results show that the covariate Age has a test P value of 0.04 in Model 2, indicating that the proportional hazards assumption is not met. However, given that the overall test P values for the two models are 1 and 0.493 respectively, both greater than 0.05, and the test P values for the target independent variable (MCMI) are also greater than 0.05, it can be concluded that the analysis models satisfy the PH assumption.

Supplementary Table 11. Table of P-values for Testing the Proportional Hazards Assumption.

| Variable | PH1 | PH2 |
| --- | --- | --- |
| MCMI | 0.5 | 0.818 |
| age | 0.44 | 0.04 |
| Education | 0.72 | 0.103 |
| maritalstatus | 0.49 | 0.791 |
| Location | 0.99 | 0.077 |
| drinking | 0.96 | 0.236 |
| smoking | 0.91 | 0.393 |
| heartproblem | 0.94 | 0.195 |
| Hypertension | 0.2 | 0.621 |
| Dyslipidemia | 0.34 | 0.534 |
| Antihypertensivedrugs | 0.2 | 0.785 |
| Lipidloweringdrugs | 0.3 | 0.499 |
| heartproblemdrugs | 0.55 | 0.499 |
| kidneydisease | 0.51 | 0.857 |
| SUA | 0.53 | 0.757 |
| CRP | 0.75 | 0.452 |
| BUN | 0.82 | 0.605 |
| Scr | 0.45 | 0.439 |
| bmi | 0.77 | 0.71 |
| GLOBAL | 1 | 0.493 |

PH1 indicates that the influence of variables including MCMI, age, gender, educational level, marital status, place of residence, smoking and drinking history, hypertension, dyslipidemia, heart disease, chronic kidney disease history, use of antihypertensive drugs, use of lipid-lowering drugs, and drug treatment for heart disease, BMI, CRP, SUA, BUM, and Scr on the incidence of stroke varies over time. PH2 assumes that the influence of these variables on mortality varies over time. When the test P > 0.05, it can be considered that the analysis model satisfies the PH assumption.

Supplementary Table 12. Three Competitive Risk Models for Stroke HR Based on MCMI.

|  | Model1 | | |  | Model2 | | |  | Model3 | | |
| --- | --- | --- | --- | --- | --- | --- | --- | --- | --- | --- | --- |
| Characteristic | HR | 95%CI | p-value |  | HR | 95%CI | p-value |  | HR | 95%CI | p-value |
| MCMI | 1.14 | 1.07,1.21 | <0.001 |  | 1.15 | 1.08,1.22 | <0.001 |  | 1.26 | 1.08,1.47 | 0.004 |
| MCMI4 |  |  |  |  |  |  |  |  |  |  |  |
| Q1 | Ref. | | |  | Ref. | | |  | Ref. | | |
| Q2 | 1.63 | 1.20,2.21 | <0.001 |  | 1.67 | 1.22,2.27 | <0.001 |  | 1.56 | 1.14,2.13 | 0.005 |
| Q3 | 1.78 | 1.32,2.41 | <0.001 |  | 1.88 | 1.38,2.55 | <0.001 |  | 1.53 | 1.10,2.12 | 0.011 |
| Q4 | 2.63 | 1.99,3.50 | <0.001 |  | 2.78 | 2.07,3.74 | <0.001 |  | 2.01 | 1.41,2.86 | <0.001 |

Abbreviation: MCMI: modified cardiometabolic index; HR: Hazard Ratio; CI: Confidence Interval.

Model 1: Unadjusted.

Model 2: Adjusted for age, gender, education level, marital status, place of residence, smoking and drinking history.

Model 3: Model 2 + adjusted for history of hypertension, dyslipidemia, heart disease, chronic kidney disease, use of antihypertensive drugs, use of lipid-lowering drugs, treatment with heart disease medications, body mass index, C-reactive protein, serum uric acid, blood urea nitrogen, serum creatinine.


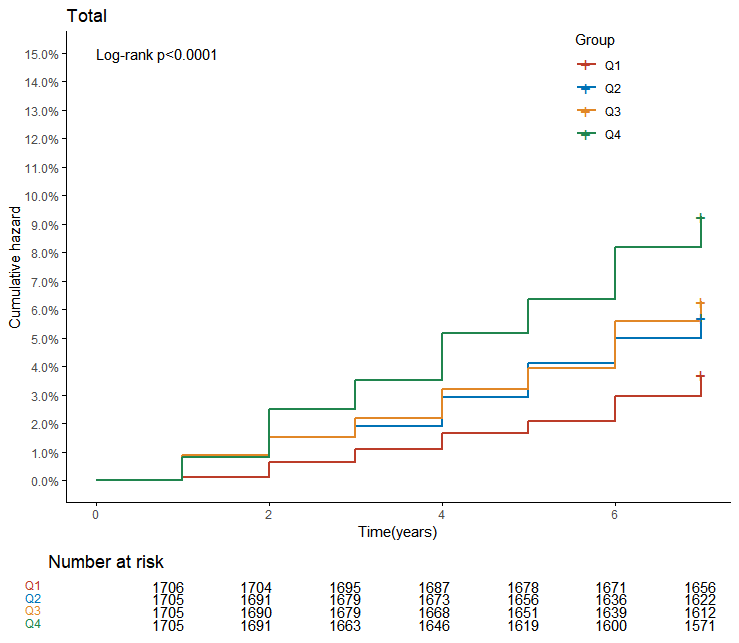


Supplementary Fig. 1. The Kaplan-Meier curve analysis, which depicts the cumulative incidence of stroke for each quartile of the MCMI (after excluding all data with missing covariates).


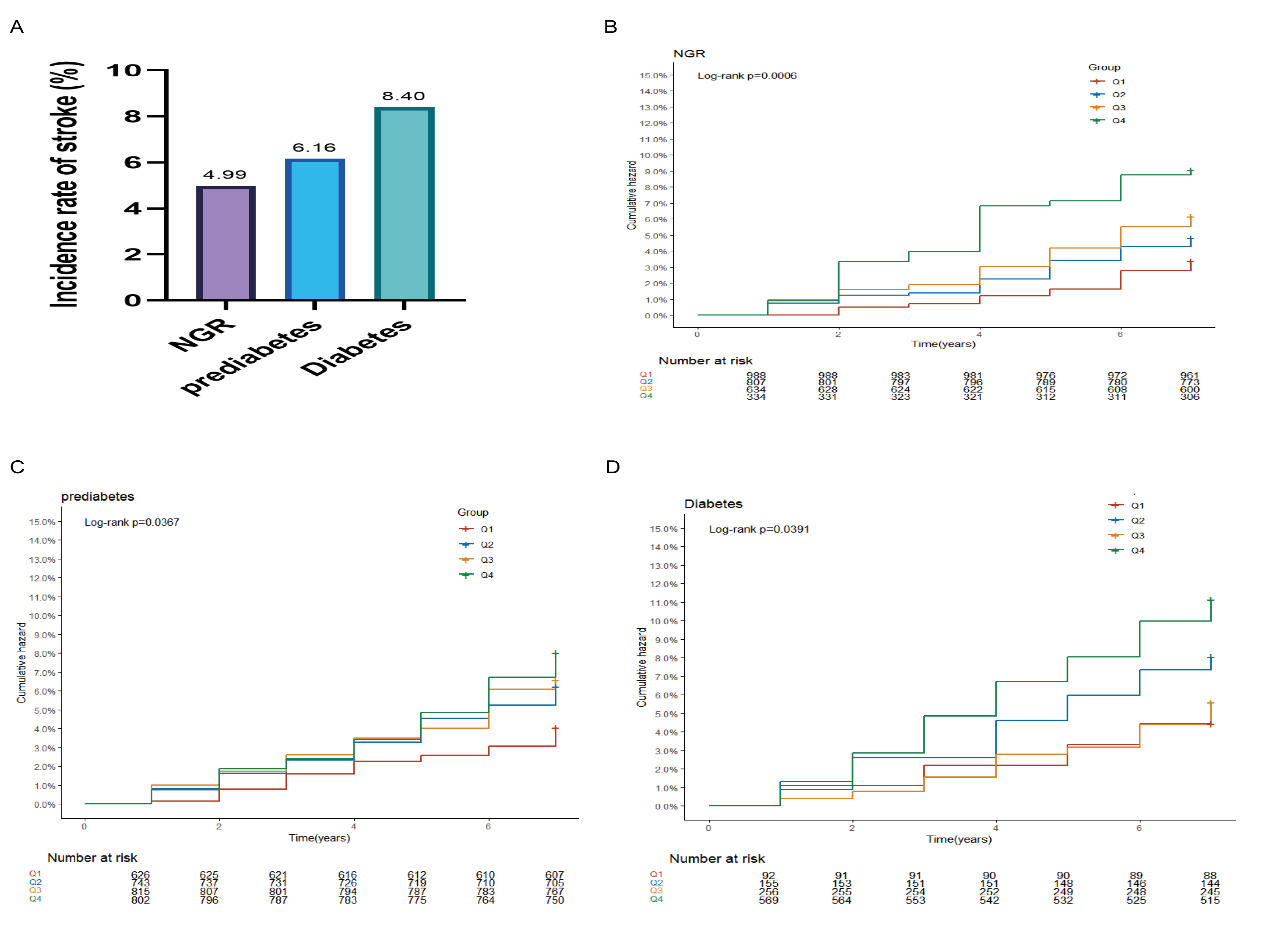


Supplementary Fig. 2. (A) Bar chart showing the incidence of stroke in different blood glucose status groups; Kaplan-Meier analysis of cumulative stroke incidence based on normal glucose regulation participants (B), prediabetic participants (C), and diabetic participants (D). (After removing all data with missing covariates)


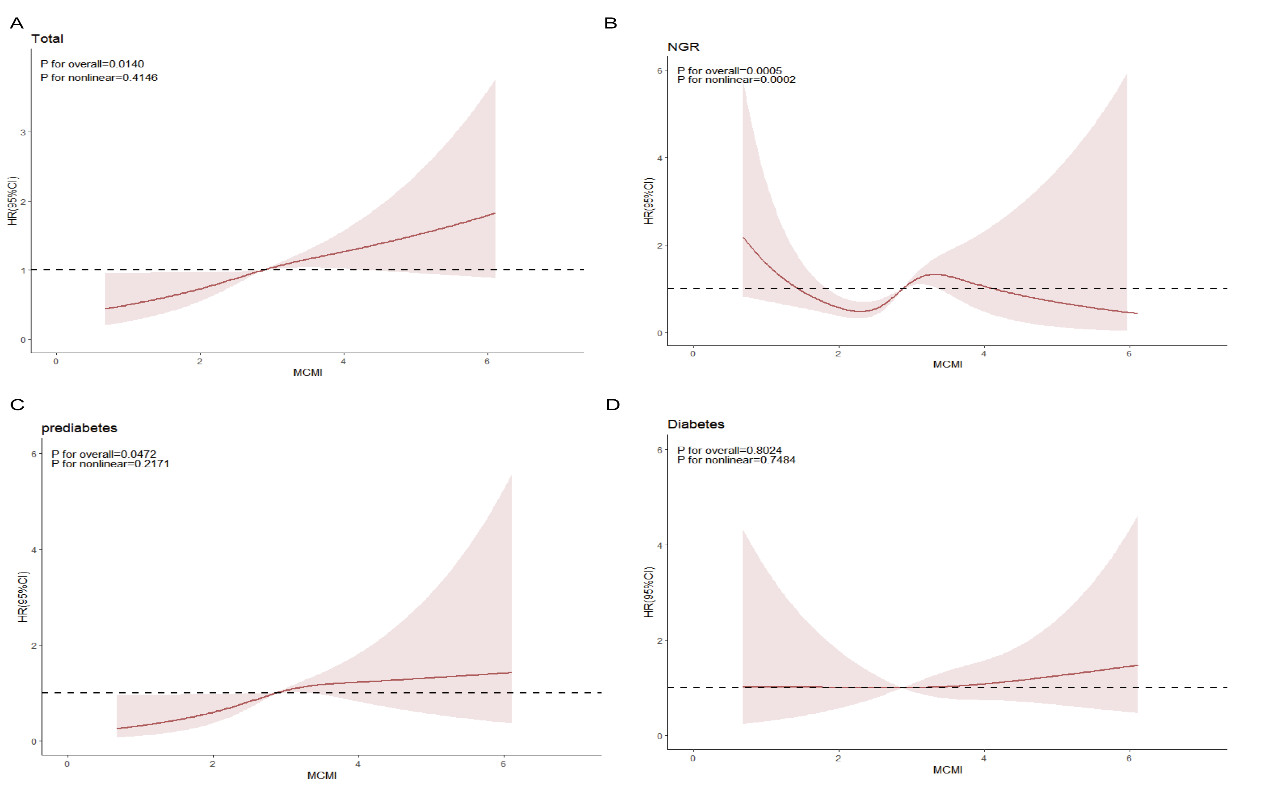


Supplementary Fig. 3. The association between the MCMI index and the risk of stroke after removing all missing data for covariates. (A) All participants; (B) NGR participants; (C) Participants with prediabetes; (D) Participants with diabetes.

The analysis was adjusted for age, gender, education level, marital status, place of residence, smoking, drinking, hypertension, dyslipidemia, heart disease, history of chronic kidney disease, use of antihypertensive drugs, use of lipid-lowering drugs, heart disease treatment, BMI, CRP, SUA, BUM, and Scr.


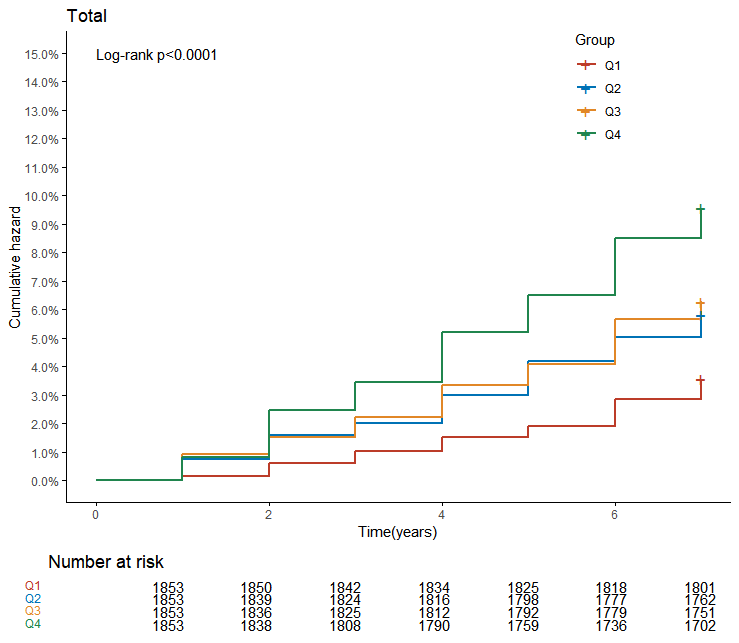


Supplementary Fig. 4. The Kaplan-Meier curve analysis, which depicts the cumulative incidence of stroke for each quartile of the MCMI (after excluding the data of deceased individuals).


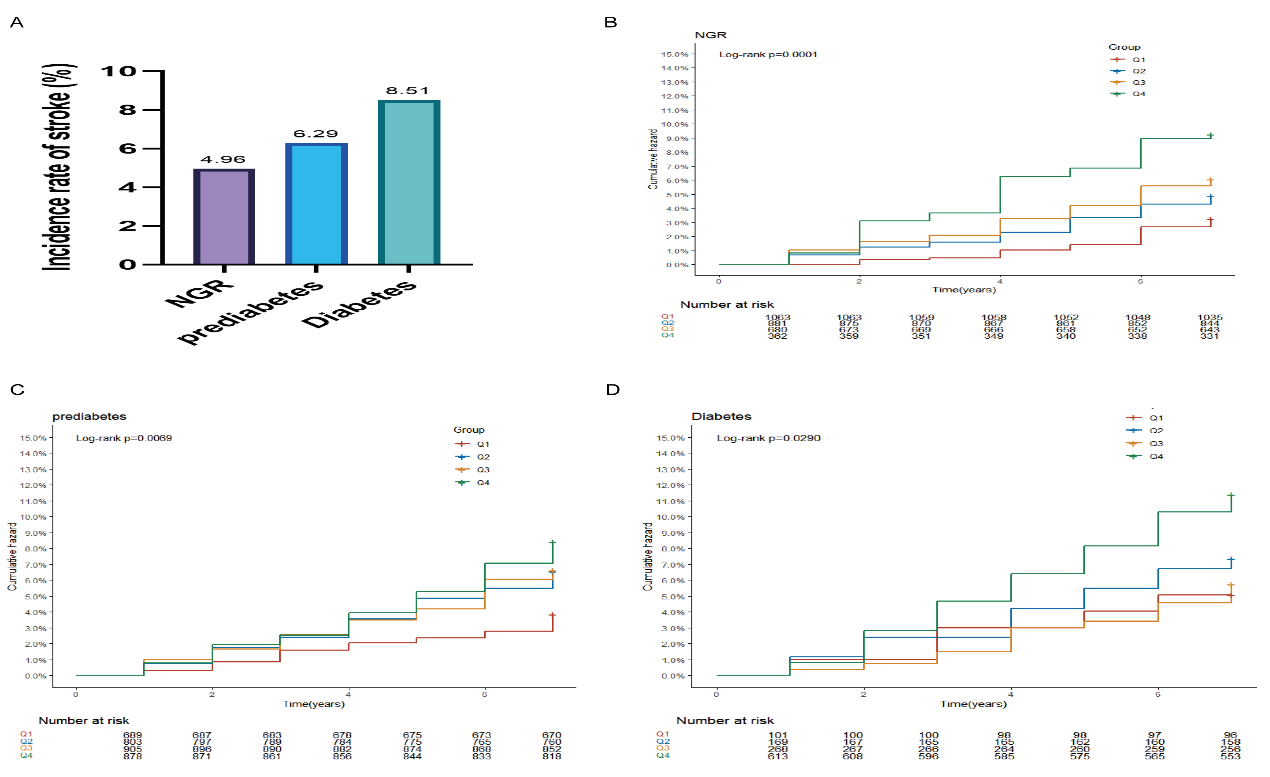


Supplementary Fig. 5. After removing the data of deceased individuals (A) The bar chart shows the stroke incidence rates in different blood glucose status groups; (B) Kaplan-Meier analysis of cumulative stroke incidence rates based on NGR participants, (C) for pre-diabetic participants, and (D) for diabetic participants.


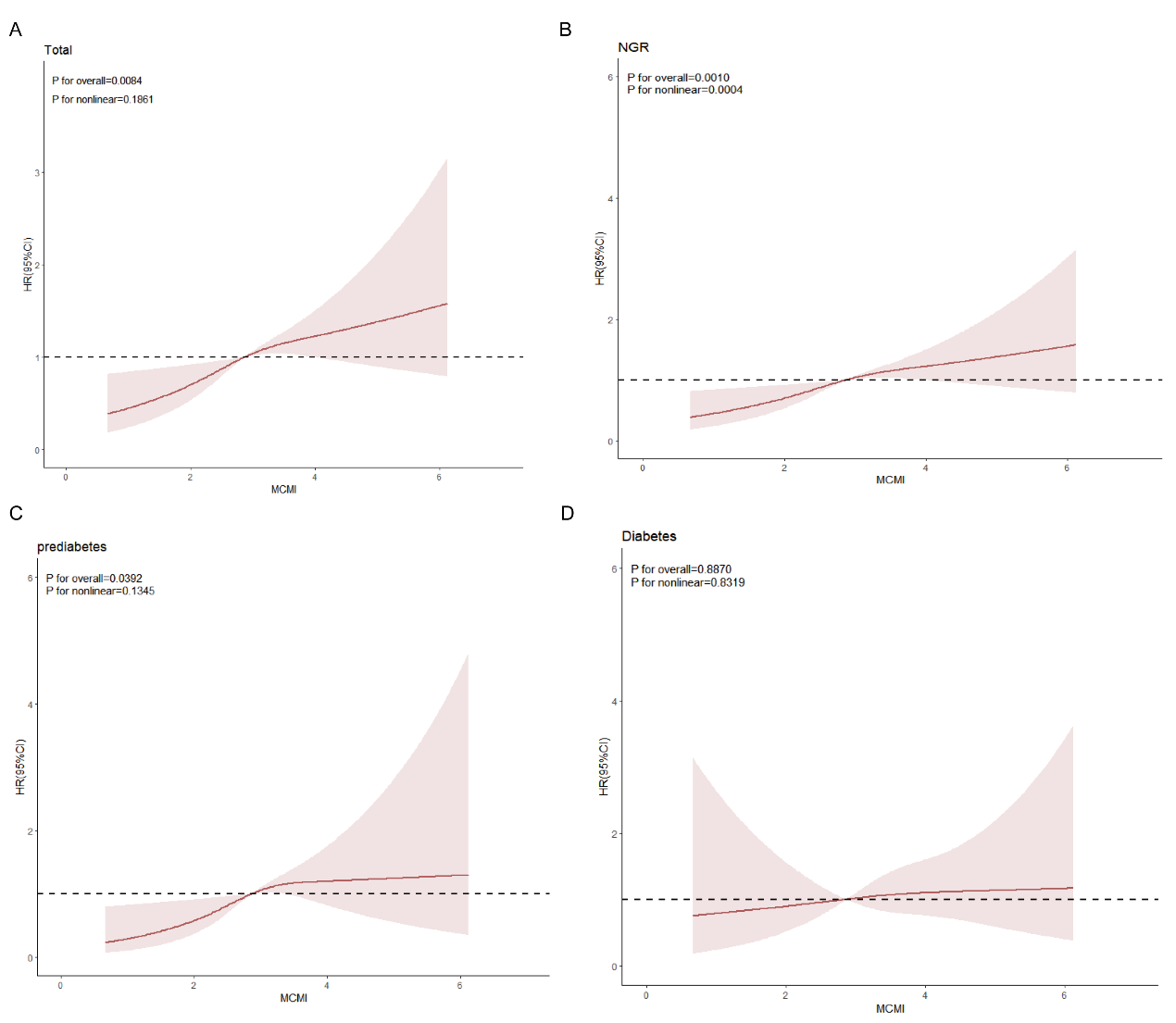


Supplementary Fig. 6. After removing the data of deceased individuals, the RCS analysis of the MCMI index and the risk of stroke was conducted. (A) Total participants; (B) NGR participants; (C) Participants with prediabetes; (D) Participants with diabetes.

The analysis was adjusted based on age, gender, education level, marital status, place of residence, smoking, drinking, hypertension, dyslipidemia, heart disease, history of chronic kidney disease, use of antihypertensive drugs, use of lipid-lowering drugs, heart disease treatment, BMI, CRP, SUA, BUM, and Scr.


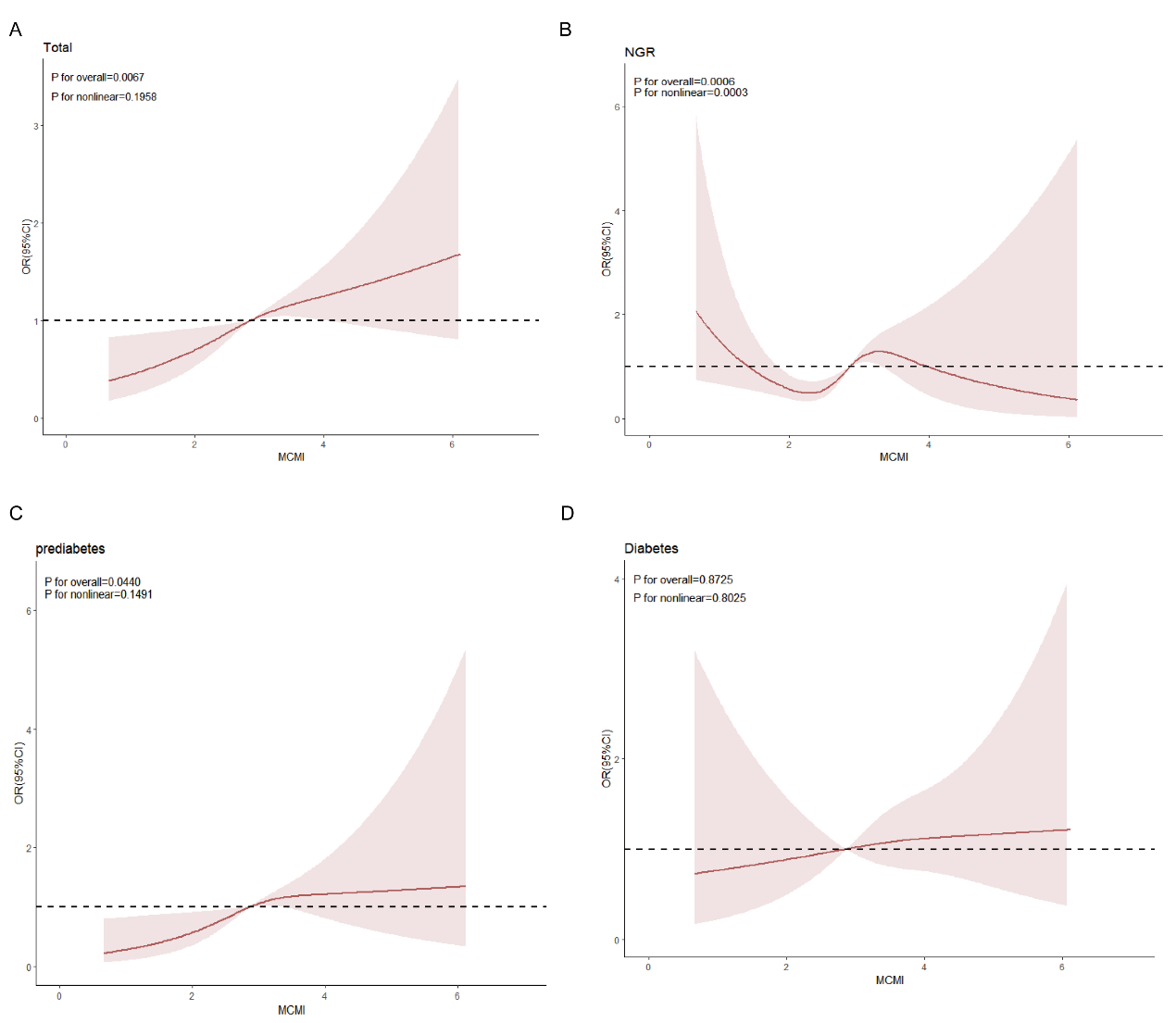


Supplementary Fig. 7. Logistic regression and RCS analysis to examine the association between the MCMI index and the risk of stroke. (A) Total participants; (B) NGR participants; (C) Participants with prediabetes; (D) Participants with diabetes.

Adjustments were made based on age, gender, education level, marital status, place of residence, smoking, drinking, hypertension, dyslipidemia, heart disease, history of chronic kidney disease, use of antihypertensive drugs, use of lipid-lowering drugs, heart disease treatment, BMI, CRP, SUA, BUM, and Scr.


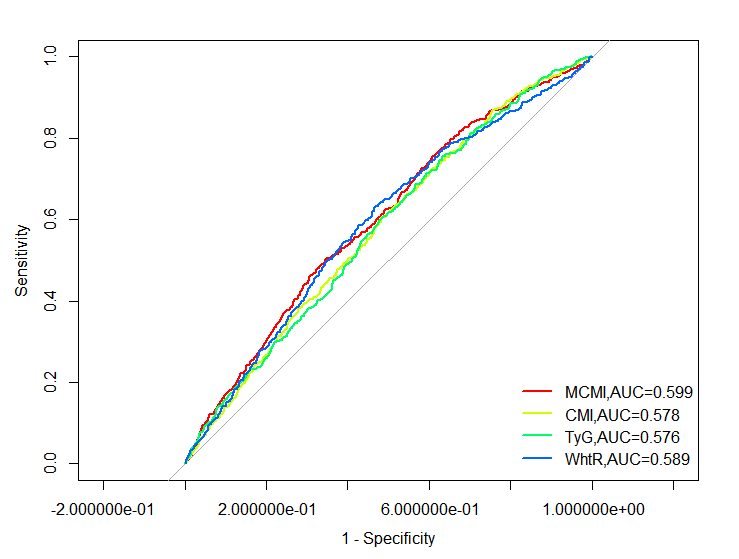


Supplementary Fig. 8. Diagnostic efficacy comparison chart.

The diagnostic performance of the MCMI, CMI, TyG index, and WHtR for the identification of new strokes is shown. The MCMI has the highest AUC for identifying strokes, with a value of 0.599, which is superior to those of the CMI (AUC = 0.578), TyG index (AUC = 0.576), and WHtR (AUC = 0.589).
